# Total Knee Arthroplasty with Intra-Articular Resection of Bone for Knee Arthritis Secondary to Malunion of a Tibial Shaft Fracture: A Radiological Evaluation of Correction of the Tibial Deformity

**DOI:** 10.1155/2021/6970591

**Published:** 2021-03-18

**Authors:** Jun-Wen Wang, Guan-Fang Chen, Hsin-Nung Shih, Shih-Hsiang Yen, Po-Chun Lin

**Affiliations:** ^1^Department of Orthopaedic Surgery, Kaohsiung Chang Gung Memorial Hospital and Chang Gung University, College of Medicine, Taiwan; ^2^Kaohsiung Municipal Min-Sheng Hospital, Taiwan; ^3^LinKou Chang Gung Memorial Hospital, Taiwan

## Abstract

This retrospective study was aimed to evaluate the clinical outcome and the extent of correction of the tibial deformity by a radiological evaluation following total knee arthroplasty (TKA) combined with intra-articular bone resection, in patients with knee arthritis and ipsilateral malunited tibial fractures. Fifteen patients (15 knees) with severe arthritis of the knee and extra-articular malunion of the tibia were treated using TKA with intra-articular bone resection. The extra-articular deformities in the coronal plane were 10 tibia vara (mean 15°, range 9°-30°), 4 tibia valgum (mean 12°, range 6°-20°), and one double deformity in the tibial shaft. The follow-up duration was 84 months (24–240). At the last follow-up, the mean Knee Society knee and function scores had improved, respectively (*p* = 0.001). The mean arc of knee motion improved from 97° preoperatively to 118.3° at the last follow-up (*p* < 0.001). The mean mechanical axis improved from a preoperative 15.5° to 1.5° of varus (*p* = 0.013). Excluding the patient with a double tibial malunion, in the 10 patients with varus tibial angulations, the tibia vara had improved from 15° preoperatively to 2.6° (*p* = 0.005). There were no observed complications except for one with a postoperative deep infection. In conclusion, our results indicated that TKA with intra-articular resection of the bone is an effective procedure for the treatment of severe arthritis of the knee with extra-articular malunion of the tibia in the coronal plane (≤30° of varus; ≤20° of valgus).

## 1. Introduction

Degenerative arthritis of the knee may result from a previous malunited fracture of the tibia outside the knee joint. Long-standing deformity of the tibia may lead to the development of degenerative arthritis due to abnormal excessive loading on the ipsilateral knee joint [1]. A controversial issue regarding treatment of this particular condition is whether to perform corrective osteotomy, either simultaneously or in a staged procedure [2, 3], or not to perform corrective osteotomy of the malunion when total knee arthroplasty (TKA) is considered [4, 5]. Combined TKA and high tibial osteotomy (HTO) in one stage have been advocated by some authors, which achieved satisfactory mechanical alignment with limited release of medial soft tissues [6]. The advantage of corrective osteotomy of the malunion is that it restores the normal tibial alignment, which is beneficial for performing TKA. A combined TKA and HTO procedure may be feasible for the treatment of a malunited tibial condylar fracture; however, in cases of angular deformity of the tibial diaphysis, the results of HTO may be different. Substantial complications, including intraoperative tibial plateau fractures, nonunion, infection of the osteotomy, and aseptic loosening of the TKA requiring revision surgery, have been reported [2, 3, 7, 8]. Furthermore, a less satisfactory function score of the knee may result due to delayed rehabilitation following simultaneous osteotomy and TKA [2, 3]. We previously reported a promising outcome of primary TKA with intra-articular resection of the tibia condyle to correct an extra-articular deformity of the tibia of <30° varus in the coronal plane [5]. In that study, the success of the outcome was based upon clinical improvement of Knee Society knee and function score as well as radiological improvement of knee alignment in the coronal plane by measuring mechanical axis. However, the extent of correction of the tibial deformity in the coronal or sagittal planes was not measured [6]. The aims of this study therefore were to evaluate the clinical outcome of TKA with intra-articular correction and soft-tissue balancing in patients with knee arthritis associated with tibial malunion and to examine the effect of correction of the tibial angulation following this procedure via a radiological evaluation method.

## 2. Materials and Methods

Between 1995 and 2015, 21 patients with severe knee arthritis associated with ipsilateral extra-articular deformity of the tibia due to malunion of previous fractures underwent primary conventional TKA with intra-articular resection of bone and soft-tissue balancing. For the knee to be included in the study, the extra-articular deformity of the tibia had to be malunion of the previous tibial shaft fractures with or without intra-articular involvement. The extra-articular deformity of the tibiae caused by metabolic disease or physiological bowing was not included. Of them, three patients have since died from unrelated diseases, and another three patients were lost to follow-up, which left 15 patients for clinical review. The study was approved by our institutional review board (201900934B0).

Eleven men and four women, with a mean age of 64.6 years (54–83) at the time of operation, were included in the study. The average interval between injury and index surgery was 34 years (8–56). The location of the malunion was in the upper third in eight, middle third in four, and lower third in two tibiae. One patient had a segmental fracture involving the upper and middle third of the tibia.

**Figure 1:**
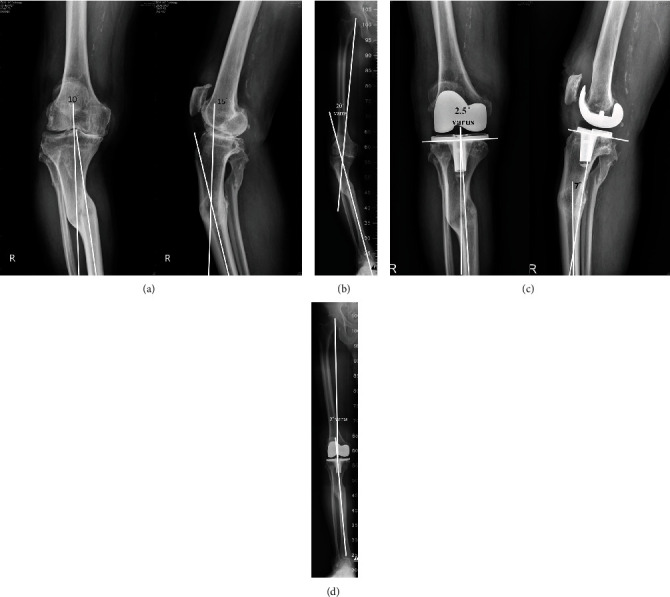
Case 9, an 83-year-old man sustained a traffic accident 40 years previously which resulted in a biplanar deformity of the right upper tibia and severe arthritis of the knee. (a) Preoperative standing radiographs of the right knee showing a malunion of the upper tibia consisting 10° of varus and 15° of antecurvatum deformities with associated severe arthritis of the knee. (b) Preoperative full-length anteroposterior radiograph of the lower extremity including hip, knee, and ankle showing a 20° of varus of the mechanical axis. (c) Standing radiographs of the knee after intra-articular bone resection of the tibia condyle and total knee arthroplasty showing measurement of the tibial deformity by the intersection angle formed between the line perpendicular to the proximal cut surface of the tibia and the long axis of the tibia distal to the deformity in the coronal and sagittal plane. It was 2.5° of varus in the coronal plane and 7° of antecurvatum in the sagittal plane. (d) The full-length radiograph of the lower extremity was made 3 years after operation showing correction of the knee alignment to a mechanical axis of 2° of varus.

The standardized X-ray protocol of our institute is described as the following: “For standing anteroposterior and lateral views of the knee, the X-ray beam is directly perpendicular to the joint line and center to the knee 1.5cm distal to the patella apex to minimize malrotation and magnification errors. The same protocol is used in the full-length anteroposterior radiograph including the pelvis to the ankle with patella facing forward and each knee in maximal extension.” The deformities of the tibia in the coronal and sagittal planes were measured by taking angulation measurements of the longitudinal axes of the proximal and distal fragments of the tibia in each plane (Figure 1). The mean angulation of the tibial deformity in the coronal plane of 10 patients was a 15° varus deformity (range 9°–30°) and in four patients was a 12° valgus deformity (range 6°–20°). A double deformity in the tibial shaft was noted in one patient, who had a 24° varus deformity in the upper tibia and a 25° valgus deformity in the tibial shaft (case 10, Table 1). In the sagittal plane, 7 patients had a recurvatum angulation (mean 8.9°, range 3°–14°), and 2 patients had an antecurvatum angulation (13° and 16°, respectively). The one patient with a double tibial deformity had a 13° recurvatum angulation in the upper third and a 10° antecurvatum angulation in the middle tibia. The remaining 5 patients did not have deformity of the tibia in the sagittal plane (Table 1). Rotational deformities of the tibia were not measured.

The mechanical axis (MA) of the knee was measured before surgery and at the latest follow-up using a full-length standing radiograph, which included the hip, knee, and ankle (Figure 1). The mean preoperative MA in the coronal plane was 15.5° varus (range, 33° varus to 18° valgus). The knee deformity, the so-called anatomical axis (AA), was measured from the angle formed between the long axis of the femur and the tibia, either proximal or distal to the level of the tibia deformity depending upon the site of malunion. The mean preoperative AA was 14.7° of varus (range, 6° varus to 25° varus) in knees with varus deformity and 21° of valgus (range, 18° valgus to 23° valgus) in knees with valgus deformity. The angulation of the tibial deformity after surgery was measured from postoperative radiographs of the tibia in the coronal and lateral planes. The tibial angulation on the coronal plane was measured from the intersection angle formed between the line perpendicular to the proximal cut surface of the tibia and the long axis of the tibia distal to the deformity. The tibial angulation on the sagittal plane after surgery was measured from the angle formed between the line parallel to the long axis of the tibial stem and the long axis of the tibia distal to the deformity on the lateral radiograph (Figure 1). The mean preoperative arc of the knee motion before surgery was 95° (range 75°–120°) (Table 1).

### 2.1. Indications for Intra-Articular Joint-Line Resection and Total Knee Arthroplasty

Our indications for intra-articular joint resection and TKA for knees with extra-articular deformity of the tibia have been reported previously [5]. A line was drawn in the medullary canal distal to the level of tibial malunion and extended to the knee joint in the coronal plane. If the line passed within the tibial condyle, TKA with intra-articular bone resection was planned; otherwise, corrective osteotomy of the tibia was indicated.

### 2.2. Surgical Technique

The knee was exposed through a conventional medial parapatellar incision. An intramedullary guidance system was used for femoral cutting, and an extramedullary guide system was employed for tibial cutting in all patients. The tibial cut line was perpendicular to the long axis of the tibia distal to the malunion site. Care was taken to limit the tibial resection up to 1 cm laterally in the tibia vara or 5 mm medially in the tibia valgum, and the remaining tibial defect was filled using bone grafts, the source of which was the resected femoral or tibial condyles. The bone-grafting procedure was performed according to the techniques described by Windsor et al. [9]. With regard to tibial cutting in the sagittal plane, we prefer to create a posterior slope of 3 to 5 degrees using the extramedullary guiding system in the setting of the posterior-stabilized TKA prosthesis.

### 2.3. Soft-Tissue Balancing

To correct both the deformity of the knee and the extra-articular deformity of the tibia in one stage by intra-articular bone resection and TKA, a greater resection of the lateral or medial condyle of the tibia was necessary, making soft-tissue balancing extremely difficult. An extensive release of the medial soft-tissue complex according to Clayton et al. [10] was required. The procedure was performed step by step, starting from the medial capsular ligaments of the knee, followed by subperiosteal release of the medial collateral ligament and pes anserinus tendons from their tibial insertions. In the case of fixed flexion contracture of the knee associated with varus deformity, the release of medial soft-tissue sleeve is continuous with semimembranosus insertion and posterior capsule. Finally, the release may include the entire medial soft-tissue cuff of the middle third of the tibial cortex in cases where correction of severe varus angulation is required (MA ≥25°, cases 1, 2, and 12). In the two cases of severe valgus deformity (AA 23° valgus, cases 3 and 7), release of the postlateral capsular ligaments from the tibial insertion, including popliteal tendon and actuate ligament, and iliotibial bands from the Gerdy's tubercle were required to achieve medial and lateral soft-tissue balancing. Compared with coronal plane deformity, there were no big issues in terms of soft-tissue release when dealing with sagittal plane deformity.

A lateral retinacular release was required in 5 patients, of whom 3 had a valgus knee deformity and 2 had a varus knee deformity. All patients underwent primary cemented TKA using a posterior-stabilized implant. The brand of knee prosthesis was Advantim (Dow Corning Wright, Arlington, TN) in 4 patients; NexGen, Legacy, High flex prosthesis (Zimmer, Warsaw, IN) in 10; and Scorpio NRG® (Stryker, Mahwah, NJ) in one patient.

Clinical evaluation using the Knee Society rating system [11] was carried out before surgery and at six weeks and three, six, and 12 months postoperatively, followed by annually thereafter. Radiological evaluation included standing AP and lateral radiographs, and a skyline view of the patella at each visit. A full-length anteroposterior radiograph of the lower extremity, including the hip, knee, and ankle, and anteroposterior and lateral radiographs of the tibia were taken at the latest follow-up to measure the postoperative alignment of the knee and the coronal and sagittal angulations of the tibia.

### 2.4. Statistical Analysis

The clinical data before and after the operation including the arc of knee motion, the Knee Society knee and function scores, the mechanical axes, and the tibial angulations in the coronal and sagittal planes were compared using the Wilcoxon signed-rank test. The level of significance was set at *p* ≤ 0.05. Statistical analysis was performed using SPSS for Windows (Statistical Package for the Social Sciences, version 22.0; SSPS Inc).

## 3. Results

The mean follow-up duration was 84 months (24 to 240). The clinical and radiological data at the last follow-up are shown in Table 2. The mean preoperative Knee Society knee score was 24.3 points (15 to 38), which improved significantly to 76.2 points (54–90) at the last follow-up (*p* = 0.001). The mean preoperative function score was 20 points (10–30), which improved significantly to 81 points (65–90) at the last follow-up (*p* = 0.001). The mean arc of knee motion improved from 97° (75°–120°) preoperatively to 118.3° (95°–130°) at the last follow-up (*p* = 0.001) (Table 3). The mechanical axis was restored from a preoperative 15.5° varus deformity (33° varus to 18° valgus) to a 1.5° varus deformity (6° varus to 2.8° valgus), an improvement of 14° (*p* = 0.013). With regard to the tibial angulation on the coronal plane, excluding one patient with a double deformity of the tibia (case 10), of the 10 patients with extra-articular varus angulations, the mean varus angulation was 2.6° (0°–7°) at the last follow-up, a significant improvement from the preoperative tibia varus angulation of 15° (9° to 30°) (*p* = 0.005). Of the remaining 4 patients with extra-articular valgus angulation of the tibia, the mean valgus angulation was 4.2° (0.2°–7.5°) at the last follow-up, showing 7.8° improvement from the preoperative tibia valgus angulation of 12° (6°–20°); however, it was not significant statistically (*p* = 0.068) (Table 3). In terms of the tibial angulation on the sagittal plane, of 7 patients with extra-articular recurvatum deformities, the mean postoperative tibial recurvatum was 1.9° (0°–5°), which had improved by 7° as compared with the preoperative extra-articular tibial recurvatum (8.9°, range 3° to 14°) (*p* = 0.017). Of the 2 patients with extra-articular antecurvatum deformities, the average postoperative tibial antecurvatum was 7.5° (7° and 8°, respectively), as compared with a preoperative extra-articular antecurvatum of 14.5° (13° and 16°, respectively). However, the significance of this improvement was not analyzed owing to the small number of cases (Table 3).

An 83-year-old man (case 9) developed congestive heart failure in the early postoperative period due to a poor cardiac status and postoperative anemia. He had a deep infection of the knee caused by *Peptostreptococcus* one month postoperative because of persistent hematoma of the knee by use of antiplatelet drugs for cardiovascular disease. Oral antibiotics therapy was given after debridement and implant retention to control periprosthetic infection. He had a poor clinical outcome at the time of the last follow-up visit. Three patients (cases 3, 4, and 7) had a less satisfactory outcome, due to subsequent cerebrovascular accident in one patient, ipsilateral fracture of the hip in another, and failed spine surgery in the third (Table 2). With the exception of the patient who suffered a deep infection, no polyethylene wear, osteolysis around the implant, or loosening of components was observed in the other 14 patients. No other complications, such as instability, periprosthetic fracture, or patellar maltracking, were observed.

## 4. Discussion

In our previous report of severe arthritis of the knee associated with extra-articular deformity of the femur or tibia, we achieved satisfactory results with intra-articular resection, soft-tissue balancing, and TKA, with no complications, after a mean follow-up duration of 38 months [5]. That study included 8 tibial deformities, with an average angulation of a 19° varus deformity in the coronal plane, with no malunions in the sagittal plane. In this study, we adhered to the principles of patient selection described in the previous study [5], as described in the indications of this procedure. All 15 knees with extra-articular tibial malunions (10 varus, 4 valgus, and one double deformities) were successfully treated with TKA with intra-articular bone resection and soft-tissue balancing, as assessed after a mean follow-up duration of 84 months (range 24–240 months), with the exception of one case that was complicated by a periprosthetic joint infection. The knee alignment improved from a preoperative 15.5° varus angle (range, 33° varus to 18° valgus) to a postoperative 1.5° varus angle (6° varus to 2.8° valgus) (*p* = 0.013) according to mechanical axis measurement.

With regard to severe knee arthritis associated with extra-articular deformity of the femur, one-stage correction of the femoral deformity by TKA with intra-articular bone resection may be beneficial for patients, in terms of the earlier rehabilitation, no additional skin incisions or complications due to corrective osteotomy of the malunion, and the satisfactory clinical outcome of this procedure [5, 12]. However, for a concomitant extra-articular deformity of the tibia, the technique of TKA with a one-stage tibial osteotomy has been advocated by some authors [6–9]. Recently, Madelaine et al. [8] reported the clinical outcome and complications of single-stage TKA and high tibial osteotomy to treat 15 knees in 12 patients, with an average follow-up duration of 78 months. The results showed improvement of clinical outcomes and the femorotibial mechanical axis; however, complications including 4 intra-articular tibial fractures and 2 revisions for nonunion at the osteotomy site were observed. Veltman et al. [7] reported their experience of one-stage TKA and corrective osteotomy for severe extra-articular deformities in 21 patients. Of them, 11 patients were treated with TKA and high tibial osteotomy for tibia vara in 4 and tibia valgum in 7. Complications included 2 deep infections, resulting in a knee arthrodesis in one and gastrocnemius transfer in another. The authors concluded that most of the patients (81%) were satisfied with the single-stage TKA and osteotomy procedure. However, patients should be informed of the higher risks of complications and failure before surgery. In this study, by radiological evaluation to measure the postoperative tibial deformity in the coronal and sagittal plane (Figure 1(c)), we experienced improved correction of tibial deformity using TKA with intra-articular bone resection and soft-tissue balancing in 10 tibia vara deformities (15° preoperatively versus 2.6° postoperatively, *p* = 0.005). With regard to 4 tibia valgum deformities, there was improvement of deformity correction (12° versus 4.2°); however, it was not significant statistically (*p* = 0.068). Our data, in addition to previous reports, showed that the use of a meticulous extensive soft-tissue release technique, intra-articular bone resection, and TKA was feasible for the treatment of severe knee arthritis with extra-articular malunion of the tibia (≤30° varus deformation and ≤20° valgus deformation). Using this technique, all complications related to tibial osteotomy are avoided, and the clinical results were similar to those of primary TKA [4, 5].

Another issue is the sagittal plane deformity of the tibial malunion. In the current study, 9 tibiae had angular deformities in the sagittal plane before surgery. The limitations of intra-articular correction of a sagittal plane deformity of the tibia during TKA have not been specified in the literature. However, our results showed that in 7 patients, the recurvatum deformity improved from a preoperative mean of 8.9° (3° to 14°) to 1.9° (0° to 5°), while in 2 patients, the antecurvatum deformity changed from 14.5° (13° and 16°) before operation to 7.5° (7° and 8°) at the last follow-up, which we consider to indicate that this procedure is beneficial in terms of maintaining knee biomechanics and for soft-tissue balancing after TKA.

We recognize there are some limitations of this study. First, the sample size was small. Because it is a rare situation in primary TKA. Most clinical reports regarding this issue usually included extra-articular deformities both in the femur and tibia. Second, the study was a retrospective review, no comparison and no post hoc power analysis, which were all related to a small sample size and long period of collection of the patients.

## 5. Conclusions

In addition to improvement of the clinical outcome, the radiological evaluation of postoperative correction of the tibial deformity demonstrated that intra-articular resection of the bone and TKA are effective in the treatment of osteoarthritis of the knee with extra-articular deformity of the tibia in the coronal plane (≤30° varus deformity and ≤20° valgus deformity).

## Figures and Tables

**Table 1:** Clinical data of 15 patients with extra-articular deformity of the tibia.

Case	Age (yrs)	Side	Gender	Interval since injury (yrs)	Site of malunion	Deformity (°)	Arc of knee motion (°)	MA (°)	AA (°)	Knee Society knee score (points)	Knee Society function score (points)	Remarks
1	64	R	M	56	Upper third	20 of varus only	15 to 90	33 of varus	20 of varus	18	15	
2	70	L	F	50	Upper third	30 of varus only	10 to 100	30 of varus	25 of varus	15	15	
3	62	R	M	40	Upper third	6 of valgus 10 of recurvatum	10 to 120	16 of valgus	23 of valgus	19	10	
4	67	R	M	37	Upper third	13 of varus 8 of recurvatum	15 to 120	13 of varus	6 of varus	32	20	
5	62	R	F	8	Middle third	9 of varus only	20 to 130	24 of varus	18 of varus	29	25	
6	54	R	F	24	Lower third	10 of varus 7 of recurvatum	0 to 120	22 of varus	12 of varus	38	30	
7	75	R	M	50	Middle third	20 of valgus 11 of recurvatum	10 to 120	18 of valgus	23 of valgus	25	20	
8	56	R	M	25	Lower third	14 of valgus only	5 to 95	18 of valgus	21 of valgus	22	20	
9	83	R	M	40	Upper third	10 of varus 15 of antecurvatum	20 to 110	20 of varus	10 of varus	24	15	
10	71	R	F	25	Upper third	24 of varus 13 of recurvatum	0 to 100	21 of varus	22 of varus	31	20	
					Middle third	25 of valgus 10 of antecurvatum						
11	64	L	M	25	Upper third	16 of varus 14 of recurvatum	20 to 120	15 of varus	14 of varus	32	25	
12	58	R	M	35	Upper third	22 of varus only	20 to 100	25 of varus	16 of varus	16	20	
13	66	R	M	30	Upper third	11 of varus 3 of recurvatum	20 to 100	10 of varus	8 of varus	23	20	
14	62	R	M	40	Middle third	8 of valgus 7 of recurvatum	10 to 100	8 of valgus	18 of valgus	17	25	
15	55	L	M	30	Middle third	9 of varus 16 of antecurvatum	5 to 110	24 of varus	13 of varus	24	20	Open tibial fracture

R: right; L: left; M: male; F: female; MA: mechanical axis; AA: anatomical axis.

**Table 2:** Postoperative results of all patients.

Case	Duration of follow-up (mths)	Arc of knee motion (°)	MA (°)	AA (°)	Tibial angulation (°)	Knee Society knee score (points)	Knee Society function score (points)	Remarks
1	240	10-105	2 of varus	5 of valgus	1 of varus only	76	85	
2	180	0-130	3 of varus	3 of valgus	2 of varus only	80	75	
3	137	0-120	2.8 of valgus	8 of valgus	2 of valgus 5 of recurvatum	66	75	
4	100	0-100	2 of varus	10 of valgus	0.5 of varus only	67	80	Ipsilat. fracture of hip
5	81	0-120	6 of varus	7 of valgus	5.5 of varus only	85	90	
6	54	0-130	2 of valgus	9 of valgus	7 of varus 2 of recurvatum	85	90	
7	70	0-130	1.6 of varus	0	7 of valgus 4 of recurvatum	64	70	
8	60	0-120	2 of varus	1 of valgus	7.5 of valgus only	85	75	
9	37	0-120	2 of varus	7 of valgus	2.5 of varus 7 of antecurvatum	54	65	Deep infection
10	32	0-115	2 of varus	5.4 of valgus		78	80	
11	30	0-130	0	6 of valgus	2 of varus 2 of recurvatum	90	90	
12	28	5-110	6 of varus	1 of varus	2.5 of varus only (upper third)	75	90	
13	26	0-125	1 of valgus	7 of valgus	0	90	90	
14	25	0-120	0	8 of valgus	0.2 of valgus only	78	85	
15	24	0-115	2.4 of varus	8 of valgus	3.3 of varus 8 of antecurvatum	70	75	

MA: mechanical axis; AA: anatomical axis.

**Table 3:** Comparison of pre- and postoperative clinical and radiological data (mean, range) in all 15 patients.

	Preoperative	Postoperative	*p* value
Arc of knee flexion (°) (*n* = 15)	95 (75~120)	118.3 (95~130)	0.001
Knee Society knee score (points) (*n* = 15)	24.3 (15~38)	76.2 (54~90)	0.001
Function score (*n* = 15)	20 (10~30)	81 (65~90)	0.001
Mechanical axis (°) (*n* = 15)	15.5 of varus (valgus 18 to varus 33)	1.5 of varus (valgus 2.8 to varus 6)	0.013
Tibial angulation^∗^			
Conoral plane (°)	Varus 15 (9 to 30 of varus) (*n* = 10) Valgus 12 (6 to 20 of valgus) (*n* = 4)	Varus 2.6 (0° to 7° of varus) (*n* = 10) Valgus 4.2 (0.2 to 7.5 of valgus) (*n* = 4)	0.005 0.068
Sagittal plane (°)			
Recurvatum (°) (*n* = 7)	8.9 (3 to 14)	1.9 (0 to 5)	0.017
Antecurvatum (°) (*n* = 2)	14.5 (13 and 16)	7.5 (7 and 8)	

^∗^Case 10 was excluded from the measurement of angulation correction because of double deformities of the tibia. ^∗^*p* value: Wilcoxon Signed Rank test.

## Data Availability

All the data that support the results can be found in the manuscript.
